# Adjunctive ketamine for sedation in critically ill mechanically ventilated patients: an active-controlled, pilot, feasibility clinical trial

**DOI:** 10.1186/s40560-021-00569-1

**Published:** 2021-08-30

**Authors:** Marwa Amer, Khalid Maghrabi, Mohammed Bawazeer, Kamel Alshaikh, Mohammad Shaban, Muhammad Rizwan, Rashid Amin, Edward De Vol, Mawadah Baali, Malak Altewerki, Mehreen Bano, Fawziah Alkhaldi, Sanaa Alenazi, Mohammed Hijazi

**Affiliations:** 1 Pharmaceutical Care Division, King Faisal Specialist Hospital and Research Center, (MBC # 11), PO Box 3354, Riyadh, 11211 Kingdom of Saudi Arabia; 2 College of Medicine, Alfaisal University, Riyadh, Saudi Arabia; 3 Department of Critical Care Medicine, King Faisal Specialist Hospital and Research Center, Riyadh, Saudi Arabia; 4 Biostatistics, Epidemiology & Scientific Computing Department, King Faisal Specialist Hospital and Research Center, Riyadh, Saudi Arabia; 5 Department of Neurosciences, Residency Training Program, King Faisal Specialist Hospital and Research Center, Riyadh, Saudi Arabia; 6 Departments of Nursing, King Faisal Specialist Hospital and Research Center, Riyadh, Saudi Arabia

**Keywords:** Ketamine, Critical care, Sedation, Mechanical ventilation, Standard of care, ATTAINMENT, Pilot

## Abstract

**Objective:**

Ketamine has been shown to decrease sedative requirements in intensive care unit (ICU). Randomized trials are limited on patient-centered outcomes. We designed this pilot trial to evaluate the feasibility of a large randomized controlled trial (RCT) testing the effect of ketamine as an adjunct analgosedative compared with standard of care alone as a control group (CG) in critically ill patients with mechanical ventilation (MV). We also provided preliminary evidence on clinically relevant outcomes to plan a larger trial.

**Material and methods:**

Pilot, active-controlled, open-label RCT was conducted at medical, surgical, and transplant ICUs at a large tertiary and quaternary care medical institution (King Faisal Specialist Hospital and Research Center, Saudi Arabia). The study included adult patients who were intubated within 24 h, expected to require MV for the next calendar day, and had institutional pain and sedation protocol initiated. Patients were randomized in a 1:1 ratio to adjunct ketamine infusion 1–2 μg/kg/min for 48 h or CG alone.

**Results:**

Of 437 patients screened from September 2019 through November 2020, 83 (18.9%) patients were included (43 in CG and 40 in ketamine) and 352 (80.5%) were excluded. Average enrollment rate was 3–4 patients/month. Consent and protocol adherence rates were adequate (89.24% and 76%, respectively). Demographics were balanced between groups. Median MV duration was 7 (interquartile range [IQR] 3–9.25 days) in ketamine and 5 (IQR 2–8 days) in CG. Median VFDs was 19 (IQR 0–24.75 days) in ketamine and 19 (IQR 0–24 days) in the CG (*p* = 0.70). More patients attained goal Richmond Agitation–Sedation Scale at 24 and 48 h in ketamine (67.5% and 73.5%, respectively) compared with CG (52.4% and 66.7%, respectively). Sedatives and vasopressors cumulative use, and hemodynamic changes were similar. ICU length-of-stay was 12.5 (IQR 6–21.2 days) in ketamine, compared with 12 (IQR 5.5–23 days) in CG. No serious adverse events were observed in either group.

**Conclusions:**

Ketamine as an adjunct analgosedative agent appeared to be feasible and safe with no negative impact on outcomes, including hemodynamics. This pilot RCT identified areas of improvement in study protocol before conducting a large, adequately powered, multicenter RCT which is likely justified to investigate ketamine association with patient-centered outcomes further.

*Trial registration* ClinicalTrials.gov: NCT04075006. Registered on 30 August 2019. Current controlled trials: ISRCTN14730035. Registered on 3 February 2020

**Supplementary Information:**

The online version contains supplementary material available at 10.1186/s40560-021-00569-1.

## Background

Analgo-sedation or analgesia-first sedation has gained popularity in recent years [1]. This approach has been developed to decrease sedative use, and facilitate mechanical ventilation (MV) weaning [2]. Data on ideal sedatives in intensive care unit (ICU) for mechanically ventilated, and hemodynamically unstable patients are limited. Ketamine has a favorable hemodynamic, analgesic, and adverse effect profile, making it attractive as an analgosedative agent [3, 4]. It inhibits *N*-methyl-d-aspartate (NMDA) receptors and activates opioid μ- and κ-receptors [5]. Anesthetists have long used ketamine for acute and chronic pain, procedural sedation, and rapid sequence intubation. It has also been used in postoperative pain control in surgical and trauma patients (as part of multimodal opioid-sparing analgesia in enhanced recovery after surgery), status asthmatics, status epilepticus, alcohol withdrawal, and agitation [6, 7].

Ketamine does not appear to have potential side effects of nonsteroidal anti-inflammatory drugs or opioids negative effects on μ receptors of gastrointestinal tract associated with ileus [8–10]. Studies to control acute pain in traumatic rib fractures of severely injured individuals at sub-anesthetic doses resulted in reduction of pain scale score and morphine-equivalent dose [11, 12]. Its use has been extended during coronavirus disease (COVID-19) pandemic due to a shortage of other sedatives to keep patients on MV comfortable and synchronous [13, 14]. Ketamine has not been associated with chest wall rigidity precipitating insufficient ventilation, which has occasionally been described with fentanyl [15]. Additionally, propofol and dexmedetomidine-associated hypotension may necessitate vasopressor support which may exclude patients from qualifying for COVID-19 antiviral medication (remdesivir), making ketamine an attractive alternative [16].

There is an increasing body of literature on ketamine use at ICU to reduce sedative requirements and maintain patients within target Sedation–Agitation Scale goal [17–20]. However, evidence provided in Pain, Agitation–Sedation, Delirium, Immobility, and Sleep Disruption (PADIS) guideline supporting its use in mechanically ventilated patients was insufficient due to limited number of randomized controlled trials (RCTs) [1, 21]. A trial by Guillou et al. showed a reduction in opioid consumption with low-dose ketamine infusion for 48 h [22]. However, patients in this trial underwent postoperative abdominal surgery and were able to use patient-controlled analgesia. It is difficult to extrapolate these findings to mechanically ventilated patients who are unable to self-report pain and have a higher severity of illness. Data on whether ketamine affects patient-centered outcomes and its safety in RCTs for critically ill patients with MV, as compared with standard of care, are unclear and have been identified as a research priority. Accordingly, we evaluated the feasibility of an analgosedative adjunct ketamine infusion in mechanically ventilated ICU patients (ATTAINMENT trial) compared to standard of care alone as a control group (CG) using an external, pilot clinical trial design. We also provided preliminary evidence on clinically relevant outcomes for the larger upcoming main trial. We hypothesized that if the study is feasible, then the main, future trial could be conducted to examine ketamine effect on patient-centered outcomes such as duration of MV with an acceptable safety profile compared to CG.

## Materials and methods

This was an investigator-initiated, pilot, single-center, parallel-group, open-label RCT (Registered with ClinicalTrials.gov: NCT04075006, current controlled trials: ISRCTN14730035, and Saudi Food and Drug Authority 19063002). The study was approved by King Faisal Specialist Hospital and Research Center (KFSH&RC) Institutional Review Board (IRB) (Riyadh, SA, IRB# 2191187) and full study protocol was published previously [23]. The trial was conducted according to CONSORT guidelines for pilot and feasibility trials and reported according to pilot study checklist [24]. Participants were recruited from KFSH&RC, a major referral center that provides tertiary and quaternary care.

Patients were eligible if they were admitted to any of three adult ICUs (medical, surgical, and transplant ICU), intubated within previous 24 h and expected to continue on MV next calendar day, initiated on institutional pain and sedation protocol, and no objection from ICU attending or primary treating team. Recruitment began in September 2019 and was completed in November 2020. Patients were excluded if they had history of dementia or psychiatric disorders, or were comatose on admission due to hepatic encephalopathy. Full inclusion and exclusion criteria are detailed in Additional file 1: Table S1. Our research coordinators, along with local principal investigators screened patients for eligibility by using an electronic screening form in Research Electronic Data Capture (REDCap). Once eligibility criteria were met, informed consent was obtained. Given the need to enroll patients in expedited manner within 24 h window, verbal consent from surrogate decision-maker (SDM) was allowed and documented in electronic medical records (EMR). Written consent was obtained as soon as SDM became available.

### Randomization procedure and treatment allocation

Patients were randomized in 1:1 allocation using a computer-generated, pre-determined randomization list created by an independent biostatistician; no stratification was performed. Group allocation was concealed until after randomization. Investigators were masked to outcomes data during the trial. Although this was an open-label, patients and families were unaware of group assignment. Additional file 1: Figure S1A, B summarizes treatment algorithm. After randomization, CG was started on KFSH&RC ICU analgesia and sedation protocol. Since it was a nurse-driven protocol, treating team placed an order regarding target Richmond Agitation–Sedation Scale (RASS), and sedatives infusions were adjusted according to RASS target by bedside ICU nurse. For those randomized to intervention group, ketamine 1–2 μg/kg/min was added as an adjunct for 48 h and could be weaned off earlier in preparation for extubation. Since this was a pilot, feasibility trial, there was no further intervention after 48 h; however, clinical outcomes and adverse events (AEs) were monitored up to day 28. Ketamine dose was reported in μg per kilogram of actual body weight per min as per institutional practice. Other aspects of care, including fluid management, vasopressors use, blood products, enteral nutrition, and early mobilization at discretion of treating team, were similar in both groups. Septic patients were managed according to latest survival sepsis campaign guidelines. Patient–ventilator asynchrony was systematically assessed and managed through inter-professional collaboration by prioritizing analgesia, and management of MV to avoid unnecessary use of neuromuscular blockers (NMB). Spontaneous awakening trial (SAT) was assessed every morning with SAT safety screen unless patients were receiving sedative infusion for status epilepticus or started on NMB post-randomization. Patients who passed SAT were immediately managed using spontaneous breathing trial protocol. Both groups received basic analgesic regimen that included paracetamol and epidural analgesia for hyperthermic intraperitoneal chemotherapy (HIPEC) patients. If delirium treatment was needed, non-pharmacological measures (reassurance or mobilization, and family support) were applied first. If this was insufficient, the protocol allowed antipsychotics use and decision was left to ICU physician.

### Outcome measures and data collection

Primary aim of this pilot trial was feasibility assessed by evaluating consent rate, recruitment success, and protocol adherence. Consent rate was deemed to be adequate if > 70% of SDMs or patients chose to participate upon being approached. Successful recruitment was defined as > 3 patients enrolled per month. Protocol adherence was defined as > 75% of protocolized intervention and assessment of protocol deviation and violation [23]. We conducted educational sessions for clinicians, nurses, and hospital pharmacies to facilitate implementation of protocol. Protocol deviation was also defined as not starting ketamine immediately after randomization (ideally within 4 h) due to pharmacy delay or non-placement of ketamine order. Feasibility thresholds (progression criteria) were pre-specified as a priori by investigators and study team after discussion with IRB. Those progression criteria were another critical decision in pilot sample size calculation. Thresholds were chosen after examining other pilot feasibility studies of complex interventions (defined as interventions with several interacting components) and summarized in Additional file 1: Table S2 [25–27].

Clinically relevant primary and co-primary outcomes for the main upcoming trial were median duration of MV, and ventilator-free days (VFDs) up to day 28. This outcome was chosen as patient-centered outcome and influenced by mortality [28]. Other clinical outcomes included the following up to 28 days: ICU and hospital length-of-stay (LOS), mortality rate, and percentage of AEs. We recorded baseline demographics, comorbidities, reasons for ICU admission, and assessed severity of illness with Acute Physiology and Chronic Health Evaluation (APACHE) II and Sequential Organ Failure Assessment (SOFA). We collected proportion and cumulative use of vasopressors, sedatives and analgesics [fentanyl, propofol, midazolam, and dexmedetomidine], and antipsychotics over 48 h post-randomization. Data on sedatives administered outside ICU during anesthesia or intraoperative were not collected. Presence of delirium was assessed using confusion assessment method for ICU (CAM-ICU), which was measured at baseline and 48 h post-randomization. If CAM-ICU scores were not available, an electronic progress note was reviewed to detect any evidence of delirium. Hemodynamic parameters [heart rate (HR) and mean arterial pressure (MAP)] were collected 48 h post-randomization. Hemodynamic changes were defined as presence of tachycardia, hypertension, and hypotension. Details about variables collected and their definitions are available in Additional file 1: Table S3. Data were stored online in REDCap and data quality assessments were executed routinely.

### Statistical analysis

Following the recommended rules for pilot trial sample size calculation when standardized effect size is unknown but expected to be small, 40 participants per group was recommended [29]. This sample size is considered to be sufficient assuming a protocol adherence of at least 75% to estimate proportion within 10% of true rate with 95% confidence. Details on statistical plan were published previously [23]. Statistician was blinded to group allocation and performed statistical analyses using R statistical software Version 3.5.0 (R Foundation, Vienna, Austria). Exploratory clinical outcomes analysis included all patients who were enrolled, randomly assigned, and received at least one dose of study medication, constituting modified intention-to-treat (mITT) population. Categorical variables were summarized as counts and percentages. Continuous variables were summarized using either mean ± SD or median and interquartile range (IQR), according to normality testing (using Shapiro–Wilk test and histograms). Chi-square test was used to compare categorical variables. Unpaired *t*-test or Mann–Whitney test was used to compare continuous variables. Sensitivity analysis for sedative and vasopressor requirements, excluding patients started on NMB post-randomization, was conducted. We also performed an additional post hoc sensitivity analysis in per-protocol population, defined as mITT population after exclusion of subjects who did not complete 48 h post-randomization [27]. We ensured immediate data entry and identified missing data quickly, and issues were resolved promptly. Thus, no imputation for missing variables was done. We set statistical significance to two-sided *p* value of 0.05.

## Results

**Fig. 1:**
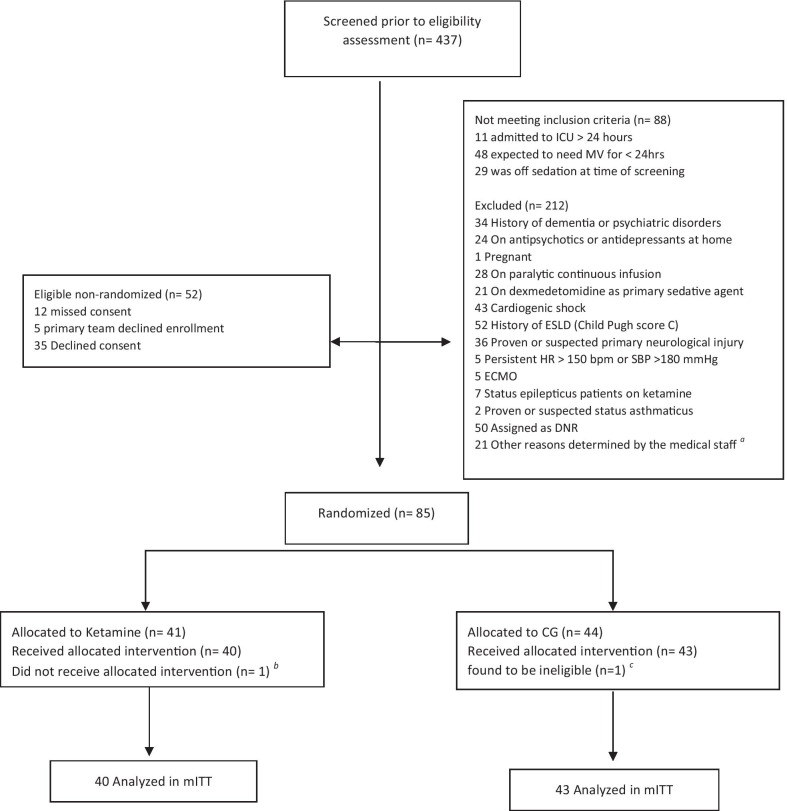
Study flowchart. ^a^3 patients had sever pulmonary hypertension, 11 had tracheostomy at baseline, 2 had intellectual disability precluded delirium assessment, 2 patients transferred from outside facility, 3 had history of substance abuse. ^b^Extubated post-randomization. ^c^Found to be on dexmedetomidine at baseline. *CG* control group (donates to standard of care), *ESLD* end-stage liver diseases, *HR* heart rate, *SBP* systolic blood pressure, *ECMO* extracorporeal membrane oxygenation, *DNR* do-not-resuscitate, *mITT* modified intention-to-treat analysis

From September 2019 through November 2020, a total of 437 patients were screened; 83 (18.9%) patients met inclusion criteria and 352 (80.5%) were excluded. Among screened patients, 88 (20.1%) did not meet eligibility criteria, mainly because they were expected to require MV for < 24 h. Among included patients, 43 were in CG and 40 were included in ketamine group in mITT analysis. Participants’ flow through the trial is shown in Fig. 1.

Baseline demographics are described in Table 1 and Additional file 1: Table S4. Median age was 60 years, with a higher proportion of males and medical ICU patients. Overall, demographic characteristics were balanced between groups, except for prevalence of chronic obstructive pulmonary disease, which was higher in CG. We included a wide variety of ICU admission diagnoses and among those randomized to ketamine, 55% had acute respiratory distress syndrome (ARDS) and about 25% were recipients of solid organ transplants or had solid malignancy. Other primary reasons for ICU admission included HIPEC (3 patients: 2 in ketamine and 1 in CG), COVID-19 pneumonia (2 patients: one in each group), and sickle cell disease (1 patient in ketamine). Ketamine-treated patients were noted to have higher median lactate level (2.2 [IQR 1.58–3.4 mmol/L] *p* = 0.004). Median number of hours of ICU admission before study enrollment was 13 h (IQR 6–21.15) in CG and 15 h (IQR 12–21) in ketamine (*p* = 0.17). Post-randomization, NMB was initiated in 12.5% of ketamine-treated patients compared to 4.65% in CG (*p* = 0.25).

**Table 1:** Demographic and baseline characteristics

Variables	All (*N* = 83)	CG (*N* = 43)	Ketamine (*N* = 40)	*p*
Age, years	61 (44.5–71)	61 (47.5–70)	59 (40.5–73)	0.61
Male, *N* (%)	51 (61.4)	25 (58.1)	26 (65.0)	0.68
Weight, kg	65 (50.7–73.2)	61.8 (47.5–69.4)	67.5 (51.9–81.2)	0.09
ICU type, *N* (%)				0.74
Medical	40 (48.2)	19 (44.2)	21 (52.5)	
Surgical	22 (26.5)	12 (27.9)	10 (25)	
Transplant	21 (25.3)	12 (27.9)	9 (22.5)	
The primary reason for ICU admission, *N* (%)				
Sepsis or septic shock	25 (30.1)	14 (32.6)	11 (27.5)	0.79
Acute respiratory distress syndrome	50 (60.2)	28 (65.1)	22 (55)	0.47
Cardiovascular	8 (9.64)	3 (6.98)	5 (12.5)	0.47
Gastrointestinal	6 (7.23)	5 (11.6)	1 (2.5)	0.20
Neurological	10 (12)	5 (11.6)	5 (12.5)	1
Trauma	2 (2.41)	1 (2.33)	1 (2.5)	1
Comorbidities, *N* (%)				
COPD	6 (7.23)	6 (14)	0 (0)	0.03
Mild liver dysfunction [Child–Pugh score A, B]	9 (10.8)	6 (14)	3 (7.5)	0.49
Diabetes	28 (33.7)	17 (39.5)	11 (27.5)	0.35
CKD	16 (19.3)	9 (20.9)	7 (17.5)	0.91
Solid malignancy	24 (28.9)	14 (32.6)	10 (25)	0.61
Hematological malignancy	14 (16.9)	4 (9.3)	10 (25)	0.11
Recipient of solid organ transplantation	21 (25.3)	11 (25.6)	10 (25)	1
HSCT	7 (8.43)	3 (6.98)	4 (10)	0.71
HIV/AIDS	1 (1.2)	0 (0)	1 (2.5)	0.48
Hypertension	24 (28.9)	11 (25.6)	13 (32.5)	0.65
Neuromuscular blockers post-randomization, *N* (%)	7 (8.43)	2 (4.65)	5 (12.5)	0.25
SOFA score	8 (5–10)	8 (6–9)	8 (5–10)	0.87
APACHE II	20 (13–26)	19 (14–25)	20.5 (13–26.75)	0.83
Lactate at baseline, mmol/L	1.8 (1.2–3.05)	1.4 (1–2.3)	2.2 (1.58–3.4)	0.004
PO_2_/FiO_2_ ratio	152 (94.1–294)	144 (88.9–263)	156 (99.2–314)	0.77
PRE-DELIRIC score^a^ (%)	20 (12–33)	20 (12–36)	20 (13–28)	0.68
Heart rate (HR), beats/min	93 (80–106)	91 (79–105)	93.5 (81.5–106)	0.48
Mean arterial pressure (MAP), mmHg	77 (69–89.5)	77 (69.5–88.5)	76.5 (65.5–91)	0.87

Data presented as *n* (%), mean ± sd, or median (interquartile range)

*APACHE II* Acute Physiology and Chronic Health Evaluation II, *CKD* Chronic kidney disease, *CG* control group (donates to standard of care), *COPD* chronic obstructive pulmonary disease, *FiO*_*2*_ fraction of inspired oxygen, *HR* heart rate, *HIV/AIDS* human immunodeficiency virus infection and acquired immunodeficiency syndrome, *HSCT* hematopoietic stem cell transplantation, *MAP* mean arterial pressure, *PaO*_*2*_ partial pressure of oxygen, *PRE-DELIRIC* prediction of delirium in ICU patients, *SOFA* Sequential Organ Failure Assessment

^a^Delirium prediction model designed for adult critical care patients 24 h after ICU admission and used to predict the factors that may influence delirium risk prior to randomization

### Feasibility outcomes

Average patients enrollment was 3–4 patients/month. Consent rate was adequate; 89.24% of SDMs or patients chose to participate when approached for consent. Recruitment rate decreased significantly during COVID-19 pandemic and was halted for 1 month. We resumed recruitment at a slower rate in March 2020, with an average of 1–2 patients/month. In total, 12% of patients were enrolled outside traditional working hours (on weekends or night shifts). This process was facilitated through close collaboration with on-call ICU physician. Two (2.4%) patients were excluded post-randomization, yielding a retention rate of 97.6%. Protocol adherence was 76% and median hours from consent or enrollment until ketamine started was 4.25 h [IQR 2.08–5.88]. Reasons of protocol non-adherence are described in Table 2.

**Table 2:** Reasons for protocol non-adherence

Variables	All (*N* = 83)	CG (*N* = 43)	Ketamine (*N* = 40)
None	63 (76)	32 (74.4)	31 (77.5)
Violated inclusion criteria	1 (1.2)	0 (0)	1 (2.5)
Violated exclusion criteria	1 (1.2)	1 (2.3)	0 (0)
Missed or discontinued trial protocol, *N* (%)	18 (21.7)	10 (23.3)	8 (20)
Reasons^a^			
Excessive sedation and patients not in target RASS, *N* (%)	2 (2.4)	0 (0)	2 (5)
Death at 48 h post-randomization, *N* (%)	4 (4.8)	2 (4.7)	2 (5)
Goal of care changed to comfort care, *N* (%)	2 (2.4)	1 (2.3)	1 (2.5)
Physician decline patient participation post-randomization, *N* (%)	1 (1.2)	0 (0)	1 (2.5)
Persistent tachycardia within the first 48 h [HR > 150 beats/min], *N* (%)	4 (4.8)	1 (2.3)	3 (7.5)
Persistent hypertension within the first 48 h [systolic blood pressure ≥ 180 mmHg], *N* (%)	1 (1.2)	0 (0)	1 (2.5)
New structural brain disease, *N* (%)	4 (4.8)	4 (9.3)	0 (0)
Unknown reason, *N* (%)	2 (2.4)	2 (4.7)	0 (0)

*CG* control group (donates to standard of care), *HR* heart rate, *RASS* Richmond Agitation–Sedation Scale

^a^Patient could have > 1 reasons

### Exploratory clinically relevant outcomes

Clinical and safety outcomes are summarized in Table 3. Median duration of MV on day 28 was 7 days in ketamine group (IQR 3–9.25) compared to 5 days in CG group (IQR 2–8). Median distribution of VFDs at day 28 was 19 days in both groups (*p* = 0.70). Median duration of ICU LOS was comparable between groups. More patients in ketamine achieved goal RASS at 24 and 48 h (67.5% and 73.5%, respectively) compared to CG (52.4% and 66.7%, respectively). Median RASS was − 2 at baseline, which gradually increased to − 1 post-randomization, indicating light sedation and ability of patients to make eye contact with verbal stimulation. Thirty-six (43.37%) patients underwent CAM-ICU assessment within 48 h post-randomization, of which 2 (5%) were positive in ketamine. Proportion of patients who did not complete 48 h of the trial was higher in ketamine (37.5%) than in CG (11.63%) and main reason was weaning off sedation in preparation for extubation. Antipsychotics were started in 3 ketamine-treated patients compared to 4 patients in CG (*p* = 1). Dexmedetomidine initiation within 48 h post-randomization was similar between groups. Higher frequency of hypersalivation and frequent suctioning was observed in CG arm. Regarding hemodynamic changes in HR and MAP at 24 and 48 h, we found no difference between groups. The 28-day mortality rate was 11 (27.5%) in ketamine compared with 14 (32.6%) in CG, *p* = 0.79. Data Safety Monitoring Committee reviewed all deaths, and all were determined to have been due to underlying disease, with participation in trial not being a contributing factor. Additional details on safety outcomes and subgroup analysis are available in Additional file 1: Table S5, Figures S2, S3.

**Table 3:** Clinical outcomes

Variables	All (*N* = 83)	CG (*N* = 43)	Ketamine (*N* = 40)	*p*
Clinical outcomes				
Liberation from MV within 28 days post-intubation, *N* (%)	52 (62.7)	27 (62.8)	25 (62.5)	1
28-day duration of MV, days	5 (2–9)	5 (2–8)	7 (3–9.25)	0.15
Duration of MV at ICU discharge/death, days	8 (3–18.5)	7 (3–13.8)	9 (3–19)	0.32
Ventilation-free days, days^a^	19 (0–24)	19 (0–24)	19 (0–24.75)	0.70
Patients at goal RASS at 24 h, *N* (%)^b^	49 (59.8)	22 (52.4)	27 (67.5)	0.24
Patients at goal RASS at 48 h, *N* (%)^c^	51 (69.9)	26 (66.7)	25 (73.5)	0.70
Patients at goal pain score at 24 h, *N* (%)^d^	80 (96.39)	41 (95.35)	39 (97.5)	1
Patients at goal pain score at 48 h, *N* (%)^d^	79 (95.2)	41 (95.3)	38 (95)	1
Discharge from ICU, *N* (%)	76 (91.6)	40 (93)	36 (90)	0.71
ICU length-of-stay, days	12 (6–22.5)	12 (5.5–23)	12.5 (6–21.2)	0.89
Hospital discharge, *N* (%)	79 (95.2)	41 (95.3)	38 (95)	1
Hospital length-of-stay, days	26 (13–39)	27 (12.5–47)	26 (15.8–38)	0.87
Safety outcomes				
CAM-ICU positive, *N* (%)^e^	2 (2.41)	0 (0)	2 (5)	0.30
Patients who did not complete 48 h of trial, *N* (%)	20 (24.1)	5 (11.63)	15 (37.5)	0.01
Hemodynamics				
HR at 24 h	92 (75.5–107)	95 (80–107)	83.5 (71.8–105)	0.11
HR at 48 h	84 (72–100)	89 (75–104)	82 (71–99)	0.31
MAP at 24 h	75 (64.5–87)	75 (62.5–86.5)	74.5 (69.5–91.5)	0.31
MAP at 48 h	77 (65–90)	76 (67.5–87)	77.5 (64–92.5)	0.50
Uncontrolled agitation, *N* (%)	10 (12.05)	4 (9.3)	6 (15)	0.51
Combative behavior to the nursing staff, *N* (%)	2 (2.41)	1 (2.33)	1 (2.5)	1
Hyper-salivation and frequent suctioning, *N* (%)	22 (26.5)	14 (32.6)	8 (20)	0.29
Antipsychotics within 48 h post-randomization, *N* (%)	7 (8.43)	4 (9.3)	3 (7.5)	1
Use of physical restraint 48 h post-randomization, *N* (%)	22 (26.5)	10 (23.3)	12 (30)	0.66
28-day mortality rate, *N* (%)	25 (30.1)	14 (32.6)	11 (27.5)	0.79

Data presented as *n* (%), mean ± sd, or median (interquartile range)

*CAM-ICU* Confusion Assessment Method for the ICU, *CG* control group (donates to standard of care), *HR* heart rate, *MV* mechanical ventilation, *MAP* mean arterial pressure, *RASS* Richmond Agitation and Sedation Scale

^a^VFDs were calculated by subtracting number of ventilation days from 28 after assigning VFD = 0 for patients who died during 28 days

^b^The RASS measures levels of consciousness (scores range from − 5 [unresponsive] to + 4 [combative]). Assessed in 82 patients at 24 h (42 CG and 40 ketamine)

^c^The RASS was assessed in 73 patients at 48 h (39 CG and 34 in ketamine)

^d^Assessment of pain was done by Critical Care Pain Observation Tool for pain (CPOT)

^e^The CAM-ICU, scores delirium as either present [positive] or not present [negative]. Assessments were done when the patient was maximally awake. If in coma, unable to evaluate

Sedation and vasopressors requirements are summarized in Table 4. There was no difference in baseline values of vasopressor and sedative requirements pre-randomization, except for amount of vasopressin which was higher in ketamine-treated patients (median 39.6, IQR 30.5–64.2 units, *p* = 0.053). Cumulative use of fentanyl and other sedatives were similar between two groups at 48 h post-randomization. Similar trends were observed for cumulative vasopressors use in mg at 48 h post-randomization. Sensitivity analysis findings, excluding those who started on NMB post-randomization or those who did not complete 48 h post-randomization, were consistent with primary analysis (Additional file 1: Tables S6–S8).

**Table 4:** Cumulative use of analgesics, sedatives, and vasopressors

	Baseline				48 h post-randomization			
	All (*N* = 83)	CG (*N* = 43)	Ketamine (*N* = 40)	*p*	All (*N* = 83)	CG (*N* = 43)	Ketamine (*N* = 40)	*p*
Fentanyl (μg)	1475 (681–2600)	1262 (488–2612)	1612 (1100–2512)	0.17	3938 (2100–6400)	3817 (2220–6140)	4400 (1588–7700)	0.67
Fentanyl (μg/kg)	23.4 (9.9–39.7)	21 (7.37–37.2)	26.4 (15.7–43.2)	0.22	66.8 (26.6–105)	63.5 (32.8–97.1)	69.6 (22.7–110)	0.69
Propofol (mg)	755 (172–1738)	780 (150–1425)	640 (215–1850)	0.37	1990 (530–3862)	2091 (492–3316)	1815 (778–4272)	0.95
Propofol (mg/kg)	10.9 (3.26–24.7)	12.7 (2.13–22.2)	10.6 (4.38–25.3)	0.63	28.4 (9.29–59)	28.4 (6.59–58.1)	28 (9.62–60.9)	1
Midazolam (mg)	5 (3–5.75)	4.75 (2–5.38)	5 (3–6)	0.54	12.5 (5.25–101)	7 (4.5–76.8)	62.8 (17.1–125)	0.11
Midazolam (mg/kg)	0.08 (0.04–0.13)	0.08 (0.04–0.13)	0.08 (0.05–0.12)	0.89	0.24 (0.1–1.25)	0.15 (0.08–0.49)	0.85 (0.26–1.74)	0.16
Dexmedetomidine (μg)					667 (357–1222)	667 (357–1222)	711 (310–1730)	0.90
Dexmedetomidine (μg/kg)					9.34 (5.33–22.8)	9.34 (5.33–22)	18 (4.67–35.5)	0.63
Norepinephrine (mg)	5.92 (2.5–12.1)	5.92 (1.82–10.7)	6.35 (3.53–14.2)	0.38	9 (4.92–28)	8.63 (6.13–26)	9.37 (4.4–28.4)	0.89
Epinephrine (mg)	1.17 (0.43–1.37)	1.24 (0.53–1.5)	0.81 (0.47–1.15)	0.36	6.09 (2–13.1)	29.2 (29.2–29.2)	4.04 (1.88–11.35)	0.16
Phenylephrine (mg)	0.45 (0.3–1.1)	0.45 (0.3–1.3)	0.45 (0.3–0.7)	0.78	0.60 (0.21–46.8)	36 (0.5–57.6)	0.45 (0.17–43.9)	0.52
Dopamine (mg)	133 (70.6–203)	133 (110–156)	149 (86.2–213)	1	563 (490–676)	563 (482–619)	602 (546–659)	0.56
Vasopressin (units)	18.2 (11.4–27.9)	12 (9.6–15)	39.6 (30.5–64.2)	0.05	70.8 (30–91.6)	24 (21.6–82.8)	81.6 (60–89.6)	0.20

Data presented as median (interquartile range)

Cumulative use at baseline donates to total amount of analgesics, sedatives, and vasopressors from ICU admission till the time of randomization. Cumulative use at 48 h donates to total amount of analgesics, sedatives, and vasopressors from time of randomization to 48 h thereafter

*CG* control group (donates to standard of care)

## Discussion

This pilot RCT explored the feasibility, and informs the design of a larger, well-designed, phase III RCT to investigate ketamine effect on clinically relevant outcomes in ICU patients with MV. Achieving our threshold of recruitment and consent rate demonstrated that the trial is feasible and acceptable to clinicians, patients, and families. Adherence rate was acceptable (achieving > 75% in 76% of total cohort). Barriers were faced during COVID-19 pandemic due to difficulties in continuing under lockdown conditions, infected research staff, shifting staff to cover COVID-19 ICU, and reorientation in clinical trial research towards COVID-19. We were able to improve adherence rate using strategies such as education sessions for clinical staff and routine clinical reminders, including documentation in EMR. We demonstrated that ketamine appeared to be safe, and had a positive effect on some surrogate clinical outcome as the majority of patients achieved target RASS and pain scores. There was no increase in antipsychotics or dexmedetomidine use post-randomization and no notable hemodynamic changes. Moreover, there was no increase in vasopressor requirements post-randomization despite the fact that ketamine-treated patients were sicker at baseline, as evident by higher lactate level and higher vasopressin dose at baseline. We also did not observe notable severe confusion, nightmares, emergence phenomena, or serious AEs associated with ketamine use, which is consistent with the findings reported by Perbert et al. [30]. Median duration of MV and VFDs in our cohort was consistent with that reported in MENDS2 sedation trial; adjusted median, 23.7 days in dexmedetomidine vs. 24 days in propofol [31]. Notably, the majority of our population were from medical ICU and had moderate ARDS, with median baseline PaO_2_/FiO_2_ ratio of 152. Overall, 28-day mortality rate in our cohort was 30.1% which is comparable to mortality rate in patients with severe sepsis and shock and all-cause mortality rate reported in more recent sedation trials, such as SPICE III trial (29% in dexmedetomidine and usual-care) and MENDS2 trial (38% in dexmedetomidine and propofol) [31–33].

Furthermore, proportion of patients who did not complete 48 h of the trials was higher in ketamine than CG and main reason was weaning off sedation for extubation. Such a difference is less likely attributed to variations in illness severity as randomization process ensured well-balanced baseline characteristics between groups in terms of ARDS severity, ICU admission reasons, SOFA, and APACHE II scores. This may be explained by the biological plausibility of ketamine in lowering airway resistance, preserving pharyngeal and laryngeal protective reflexes, and increasing lung compliance without causing respiratory depression in slow infusions [4]. The underlying mechanisms are not fully elucidated yet. One hypothesis is that ketamine has anti-cholinergic effect resulting in bronchodilation, which might be beneficial compared to other sedatives. However, we cannot be entirely sure that our findings are a direct consequence of ketamine rather than an independent improvement of clinical conditions or cumulative effect of concomitant therapies and co-interventions.

Ketamine opioid-sparing effect has been demonstrated in some trials and retrospective studies mainly for ICU patients admitted for postoperative reasons and trauma [20, 22, 34]. In contrast, other trials did not show opioid-sparing effect [30, 35, 36] (Additional file 1: Table S9). In our pilot, there was no difference in amount of sedatives or opioids and underlying reasons are likely multifactorial. Firstly, NMB initiation post-randomization was numerically higher in ketamine but statistically insignificant. Additionally, during COVID-19 pandemic, newly hired non-ICU nurses (to cover manpower shortage) could be unaware of study protocol. Hence, efforts to reduce concomitant sedatives or opioids with ketamine perhaps were conservative. Likewise, we hypothesized that severity of illness, insufficient power, and small ketamine dose in our pilot might provide possible additional explanations.

Our pilot trial had several strengths. Firstly, it included high rates of completed follow-up, and relatively comprehensive assessments of AEs associated with ketamine use and its impact on hemodynamic response. We believe that our results provide incremental value in understanding the effects of ketamine. Adherence to mITT principle, randomization, and blinded outcome assessors limited potential sources of bias. Moreover, our trial included diverse ICU populations and we made every effort to include patients within a narrow randomization window (within 24 h of intubation) to eliminate potential confounders with other co-interventions. Additionally, information on clinical outcomes was necessary to define clinically meaningful effect of ketamine use which will have an implication on sample size calculations for main upcoming RCT.

There are several limitations worth mentioning. This pilot trial had small sample size and was underpowered to detect true differences in clinical outcomes or subgroup analysis. This will be explored further in adequately sized definitive trial. Although our adherence rate met the feasibility threshold, it is possible to achieve high adherence rate than ATTAINMENT study achieved in this clinical population. We will consider additional strategies to increase protocol adherence for definitive trial (printed checklist of study protocol for bedside nurses, and study signs posted in patients’ rooms). Furthermore, medications administration in our pilot trial was unblinded which may have influenced overall RASS and CAM-ICU score assessment and physicians behavior. Therefore, we cannot exclude a possible bias related to open-label design. To minimize this bias, outcome adjudicators and patients and their families were blinded to treatment assignments, and study investigators remained blinded to the results until study conclusion.

This pilot trial highlighted areas of improvement in study protocol before launching a large, adequately powered, multicenter RCT which is likely justified to define ketamine role in ICU patients powered to examine patient-centered outcomes. We believe that the trial protocol could be improved by modifying the current ketamine dosing regimen. We chose ketamine dosing at 1–2 µg/kg/min because majority of ICU population included in our pilot were older (median age 61 years), with renal and hepatic dysfunction, which potentially alters metabolism and excretion of ketamine and its active metabolite, resulting in increased sensitivity to ketamine, prolonged duration, drug accumulation, and possible longer recovery [37, 38]. Moreover, the dose described here was in agreement with existing literature describing light sedation strategy and 2018 PADIS guideline recommendations [1, 13, 18]. Published data for ketamine doses showed that it can be safely titrated up to 15 µg/kg/min, as needed, to achieve desired level of analgosedation [4, 6]. Additional file 1: Figure S4 describes the proposed treatment algorithm for the definitive trial with a modified ketamine dosing regimen (titrated to effect).

Moreover, we excluded 17% of patients due to proven or suspected primary neurological injuries such as those with severe traumatic brain injury (TBI) and hydrocephalus. More recent systematic reviews of mixed acute brain populations (subarachnoid hemorrhage, tumors, and TBI) concluded that ketamine had no detrimental effect on intracranial pressure, ICU LOS, or mortality [39]. Therefore, future RCT may also consider modifying eligibility criteria to include neurocritical care patients to maximize generalizability and improve enrollment. However, this may have an implication on sample size calculation and power.

Additionally, small observational reports in burn and COVID-19 patients (5 patients) have linked ketamine chronic high doses (16–50 µg/kg/min for up to 26 days) with possible cholangiopathy. We have not observed this concern in our cohort and reasons for cholangiopathy in the aforementioned cases were multifactorial (hemodynamic instability, high positive end-expiratory pressures, reducing hepato-splanchnic blood flow, and direct viral cytopathic effect). We will consider vigilant monitoring of liver tests along with other relevant differentials for future RCT protocol to explore this further [40, 41].

We also did not collect data on frequency and duration of prone positioning for ARDS patients who were made prone, or median change in PaO_2_/FiO_2_ ratio post-randomization, limiting the ability to determine the real benefit of ketamine in oxygenation post-randomization. Although we made efforts to validate delirium diagnosis and its assessment with CAM-ICU, we had a large proportion of patients (56.6%) with un-assessed CAM-ICU. As such, we plan to include data for MV settings after randomization, CAM-ICU assessments, and other co-interventions (e.g., corticosteroids, prone positioning, and diuretics) in main future RCT study protocol. Finally, ketamine duration was limited to 48 h due to the nature of this pilot trial. This duration was chosen based on available evidence on the use of sedation in critically ill patients with MV [18, 31, 33]. We assumed that majority of patients remain on sedation and MV for 48 h. This assumption aligned with our own clinical experience at bedside, during which we have observed most of patients usually do not require sedation after 48 h and likely get extubated within this period. Considering ketamine pharmacokinetics (metabolized in liver, generating active compounds norketamine and hydroxynorketamine, and eliminated in urine with an elimination half-life of roughly 1.5–3 h), it is likely that carryover effects occurred despite study design for 48 h [37, 38]. Nevertheless, longer duration with close monitoring will be investigated in future definitive trial. Moving forward, a multicenter RCT in collaboration with Saudi Critical Care Trials group will be conducted. Data safety monitoring committee and steering committee will oversee the running of the trial and ensure overall safety of participants. An interim analysis with a pre-specified frequency will be carried out for purposes of determining futility or success of treatment and whether early stopping is appropriate. Specification of interim analysis stopping rule and sample size calculation will be reported separately in main RCT study protocol.

## Conclusions

Ketamine is a potentially attractive option for analgosedation. In our pilot trial, ketamine appeared to be safe, and feasible. However, the lessons learned from this pilot will usefully inform the design and study protocol before conducting main, adequately powered, multicenter RCT to shed light on remaining questions and investigate the association with patient-centered outcomes further. Modifying ketamine dosing regimen, some eligibility criteria, and inclusion of additional data (liver function tests, co-interventions and CAM-ICU) were identified as main goals for modifications to improve recruitment and generalizability.

## Supplementary Information

**Additional file 1.Tables S1:** Full inclusion and exclusion criteria. **Figure S1A:** Treatment algorithm for patient randomized to standard of care (Control Group; CG). **Figure S1B:** Treatment algorithm for patient randomized to ketamine. **Table S2:** Feasibility thresholds (progression criteria) and intervention stopping rule for other pilot feasibility studies with complex intervention. **Table S3:** Details of outcome variables definition. **Table S4:** Other demographic and baseline characteristics. **Table S5:** Other safety outcomes. **Figure S2:** HR and MAP at baseline, 24-hours, and 48-hours. **Figure S3:** Subgroup analysis for selected outcomes. **Table S6:** Proportion of sedatives and vasopressors. **Table S7:** Sensitivity analysis for sedatives and vasopressors requirements excluding patients started on atracurium post-randomization. **Table S8:** Post-hoc sensitivity analysis excluding patients who did notcomplete 48-hours due to extubation or sedation weaned off. **Table S9:** Ketamine studies that showed and did not show opioid-sparing effect. **Figure S4:** Proposed treatment algorithm for the definitive trial with modified ketamine dosing regimen.

## Data Availability

The datasets used and analyzed during the current study are available from the corresponding author on reasonable request.
