# Supplementary material for: Adjunctive ketamine for sedation in critically ill mechanically ventilated patients: an active-controlled, pilot, feasibility clinical trial
Source: J Intensive Care. 2021 Aug 30;9:54. doi: 10.1186/s40560-021-00569-1 (PMC8404029; doi:10.1186/s40560-021-00569-1)
Supplement: Supplementary file 1 — Additional file 1.Tables S1: Full inclusion and exclusion criteria. Figure S1A: Treatment algorithm for patient randomized to standard of care (Control Group; CG). Figure S1B: Treatment algorithm for patient randomized to ketamine. Table S2: Feasibility thresholds (progression criteria) and intervention stopping rule for other pilot feasibility studies with complex intervention. Table S3: Details of outcome variables definition. Table S4: Other demographic and baseline characteristics. Table S5: Other safety outcomes. Figure S2: HR and MAP at baseline, 24-hours, and 48-hours. Figure S3: Subgroup analysis for selected outcomes. Table S6: Proportion of sedatives and vasopressors. Table S7: Sensitivity analysis for sedatives and vasopressors requirements excluding patients started on atracurium post-randomization. Table S8: Post-hoc sensitivity analysis excluding patients who did notcomplete 48-hours due to extubation or sedation weaned off. Table S9: Ketamine studies that showed and did not show opioid-sparing effect. Figure S4: Proposed treatment algorithm for the definitive trial with modified ketamine dosing regimen. [file 40560_2021_569_MOESM1_ESM.pdf]

Additional file

Table of Content	Page
**Table S1**: Full inclusion and exclusion criteria	2-3
**Figure S1A**: Treatment algorithm for patient randomized to standard of care (Control Group; CG)	4
**Figure S1B**: Treatment algorithm for patient randomized to ketamine	5
**Table S2:** Feasibility thresholds (progression criteria) and intervention stopping rule for other pilot feasibility studies with complex intervention	6
**Table S3:** Details of outcome variables definition	7-9
**Table S4:** Other demographic and baseline characteristics	10
**Table S5:** Other safety outcomes	11
**Figure S2:** HR and MAP at baseline, 24-hours, and 48-hours	12
**Figure S3:** Subgroup analysis for selected outcomes	13-14
**Table S6:** Proportion of sedatives and vasopressors	15
**Table S7:** Sensitivity analysis for sedatives and vasopressors requirements excluding patients started on atracurium post-randomization	16
**Table S8:** Post-hoc sensitivity analysis excluding patients who did not complete 48-hours due to extubation or sedation weaned off	17-18
**Table S9:** Ketamine studies that showed and did not show opioid-sparing effect	19
**Figure S4:** Proposed treatment algorithm for the definitive trial with modified ketamine dosing regimen	20

Table S1: Full inclusion and exclusion criteria

**Inclusion criteria:**
1. Adults patients (>14 years).<br>2. Recently intubated and commenced on mechanical ventilation within the last 24-hours.<br>3. Admitted to one of the following ICUs (Medical, Surgical, transplant/oncology or COVID-19).<br>4. Expected to require MV longer than 24-hours.<br>5. Expected to be on the KFSH&RC sedation protocol.<br>6. There is no objection of the ICU attending for enrollment.
**Patients with the above inclusion criteria and has one of the following exclusion criteria were screened then excluded:**
• Patients with a history of dementia or psychiatric disorders or those on any antipsychotic or antidepressant medications at home.<br>• Pregnancy.<br>• Age < 14 years old.<br>• Expected to need MV < 24-hours.<br>• Known hypersensitivity to ketamine.<br>• Patients with expected targeted RASS score of − 5, e.g., patients on continuous infusion neuromuscular blockade.<br>• Patients on dexmedetomidine as the primary sedative prior to randomization.<br>• Patients with cardiogenic shock, acute decompensated heart failure, or myocardial infarction.<br>• History of end-stage liver failure (Child-Pugh score C).<br>• Proven or suspected primary neurological injury (traumatic brain injury, ischemic stroke, intracranial hemorrhage, spinal cord injury, anoxic brain injury, brain edema).<br>• Patients with persistent heart rate (HR) > 150 beats per minute (bpm) or systolic blood pressure (SBP) >180mmHg.<br>• Patients identified as Do Not Resuscitate (DNR) or those expected to die within 24-hours.<br>• Patients on extracorporeal membrane oxygenation (ECMO).<br>• Patients with refractory status epilepticus who are receiving ketamine infusion.<br>• Proven or suspected status asthmaticus (the dose of this indication differed from the recommended dose for analgosedation).

- Severe pulmonary hypertension.
- Tracheostomy at baseline.
- Intellectual disability that precluded delirium assessment.
- Transfer from an external facility.
- History of substance abuse.
- Situations where high blood pressure could trigger dangerous complications, such as aortic dissection.
- Repeated ICU admissions within same hospital visit.
- Those who participated in another interventional trial.

Figure S1A: Treatment Algorithm for Patient Randomized to Standard of Care (Control Group; CG)

Patient meets the randomization criteria

↓

Start analgesia: clinician's choice of opioid (Fentanyl boluses or infusion 50 - 200 µg/hr)

↓

If target RASS is not achieved (RASS ≥ 2)

↓

- Optimize fentanyl and analgesia
- Start propofol (can be given by intermittent boluses or infusion). Titrate the infusion to target RASS. Usual infusion 10-200 mg/hr
- Dexmedetomidine 0.2-1.5 µg/kg/hr can be given if extubation is expected
- Midazolam as intermittent boluses may be used for patients with severe hypotension or have other compelling indication for benzodiazepine (e.g. neuromuscular blocker infusion, status epilepticus, alcohol withdrawal)

Figure S1B: Treatment Algorithm for Patient Randomized to Ketamine

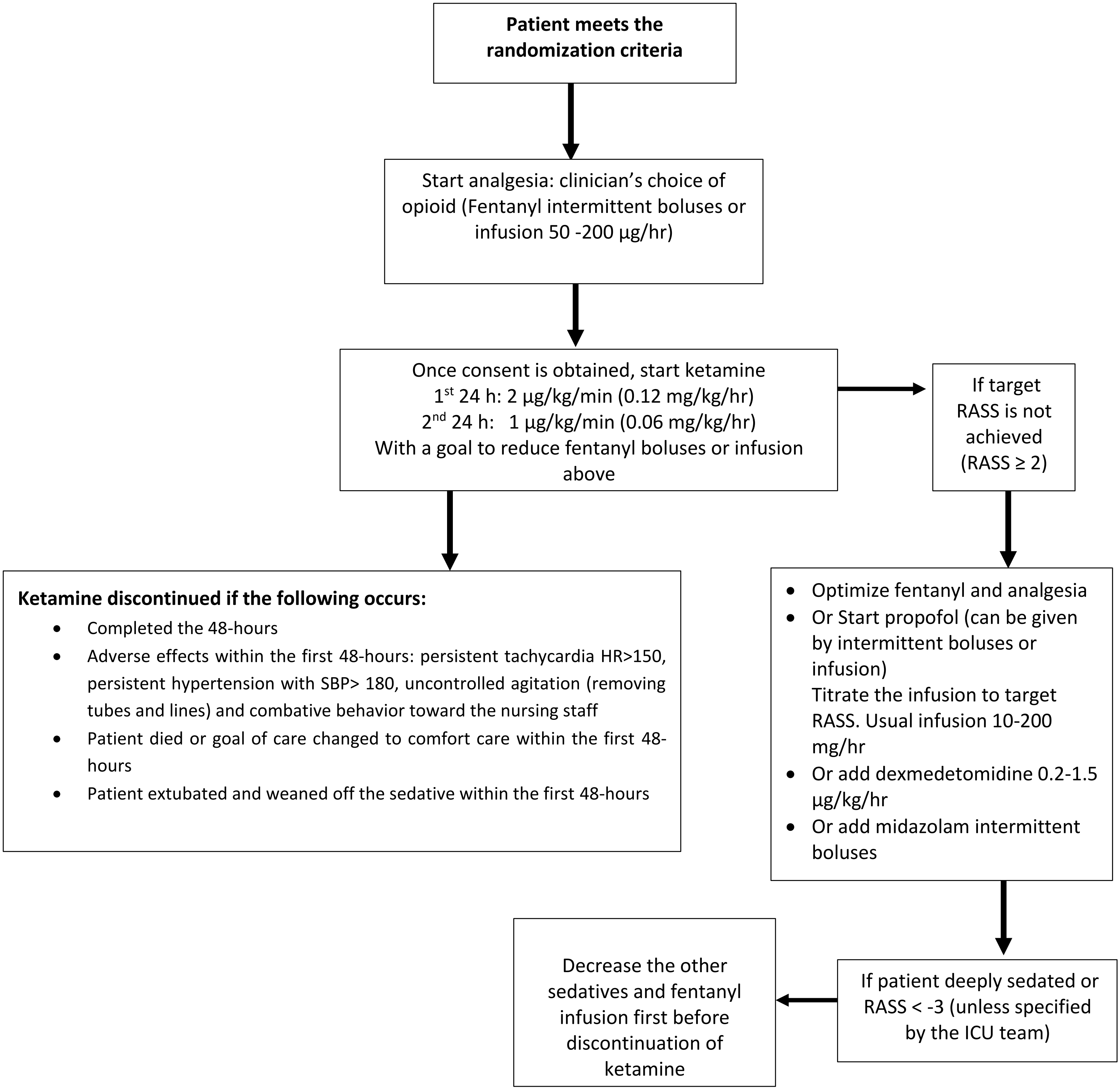

Table S2: Feasibility thresholds (progression criteria) and intervention stopping rule for other pilot feasibility studies with complex intervention [a] (1)

Pilot feasibility trial	Feasibility thresholds (progression criteria) and assessment of intervention stopping rule
Rochwerg B et al. Fluids in Sepsis and Septic Shock (FISSH): protocol for a pilot randomized controlled trial. Canadian Critical Care Trials Group (2)	Consent rate was defined as (>70%), protocol adherence was defined as receipt of study fluid for >75% of all intravenous fluids administered in the ICU excluding blood products and medication infusions
Vaara, S.T et al. . Restrictive fluid management (RFM) versus usual care in acute kidney injury (REVERSE-AKI): a pilot randomized controlled feasibility trial (3)	Protocol violations occurred in 18 (36.7%) in the RFM arm and in 5 (9.8%) in the usual care arm.<br>5 patients in the RFM arm had a protocol suspension<br>1 patient in the usual care arm had protocol suspension
Martin DS et al. A feasibility randomised controlled trial of targeted oxygen therapy in mechanically ventilated critically ill patients (4)	The protocol adherence was 73.1 in conservative O2 group and 75.2% in usual care group
Coe S et al. A protocol for a randomised double-blind placebo-controlled feasibility study to determine whether the daily consumption of flavonoid-rich pure cocoa has the potential to reduce fatigue in people with relapsing and remitting multiple sclerosis (RRMS) (5)	Successful adherence to the intervention was defined as at least 75% of the participants having completed cocoa consumption. Higher % than this is considered satisfactory. An adherence rate lower than this would require substantial changes to the intervention and require further piloting

[a] Defined as interventions with several interacting components

References:

(1) Craig P, Dieppe P, Macintyre S, Michie S, Nazareth I, Petticrew M. Developing and evaluating complex interventions: the new medical research council guidance. BMJ. 2008;337:a1655. https://doi.org/10.1136/bmj.a1655

(2) Rochwerg B, Millen T, Austin P, Zeller M, D'Aragon F, Jaeschke R, Masse MH, Mehta S, Lamontagne F, Meade M, Guyatt G, Cook DJ; Canadian Critical Care Trials Group. Fluids in Sepsis and Septic Shock (FISSH): protocol for a pilot randomised controlled trial. BMJ Open. 2017 Jul 20;7(7):e017602. doi: 10.1136/bmjopen-2017-017602.

(3) Vaara, S.T., Ostermann, M., Bitker, L. et al. Restrictive fluid management versus usual care in acute kidney injury (REVERSE-AKI): a pilot randomized controlled feasibility trial. Intensive Care Med 47, 665–673 (2021). https://doi.org/10.1007/s00134-021-06401-6

(4) Martin DS, McNeil M, Brew-Graves C, et al. A feasibility randomised controlled trial of targeted oxygen therapy in mechanically ventilated critically ill patients. Journal of the Intensive Care Society. April 2021. doi:10.1177/17511437211010031

(5) Coe S, Collett J, Izadi H, Wade DT, Clegg M, Harrison JM, et al. A protocol for a randomised double-blind placebo-controlled feasibility study to determine whether the daily consumption of flavonoid-rich pure cocoa has the potential to reduce fatigue in people with relapsing and remitting multiple sclerosis (RRMS). Pilot Feasibility Stud. 2018 Jan 23;4:35. doi: 10.1186/s40814-018-0230-7.

Table S3: Details of outcome variables definition

Severity of illness	• Estimated by Sequential Organ Failure Assessment (SOFA) score and Acute Physiology and Chronic Health Evaluation (APACHE II) score, with higher scores indicating higher severity of illness<br><br>• The Sequential Organ Failure Assessment (SOFA) is used to track organ failure in the ICU; scores range from 0 to 24, with higher scores indicating greater severity of illness<br><br>• The Acute Physiology and Chronic Health Evaluation (APACHE II) assesses the risk of death on a scale from 0 to 71, with higher scores indicating a higher risk of death.
PRE-DELIRIC<br>Delirium Risk Score	• A delirium prediction model specifically designed for adult critical care patients 24 h after ICU admission. This model was used to predict the factors that may influence delirium risk prior to randomization<br>**Formula for PRE-DELIRIC model (1)**<br>Risk of delirium = 1/(1+exp−(−6.31<br>+ 0.04 × age<br>+ 0.06 × APACHE-II score<br>+ 0 for non-coma or 0.55 for drug induced coma or 2.70 for miscellaneous coma or 2.84 for combination coma<br>+ 0 for surgical patients or 0.31 for medical patients or 1.13 for trauma patients or 1.38 for neurology/neurosurgical patients<br>+ 1.05 for infection<br>+ 0.29 for metabolic acidosis<br>+ 0 for no morphine use or 0.41 for 0.01-7.1 mg/24 h morphine use or 0.13 for 7.2-18.6 mg/24 h morphine use or 0.51 for >18.6 mg/24<br>h morphine use<br>+ 1.39 for use of sedatives<br>+ 0.03 × urea concentration (mmol/L)<br>+ 0.40 for urgent admission))<br>The scoring system's intercept is expressed as −6.31; the other numbers represent the shrunken regression coefficients (weight) of each risk factor.
Duration of MV	• Was recorded as either the number of calendar days from intubation to extubation or until ICU discharge or death, whichever occurred first.<br><br>• This outcome was chosen as a patient-centered outcome and based on the mechanistic plausibility data that showed ketamine possibly has a bronchodilatory effect and maintains respiratory drive and airway reflexes
Successful extubation	• Was defined as the absence of the need for reintubation within 48 hours.
Cumulative dose of pain and sedatives	• Reported as proportion and median dose in the first 48 h after randomization
Patients achieving the RASS goal and pain score goal	• Reported as proportion of patients achieving this goal within the first 24 and 48 h after randomization

	• The RASS is a scale used to assess the depth of sedation on a scale of − 5 to + 4, with a negative value indicating deeper sedation and positive values indicating increased agitation<br><br>• Assessment of pain was done by Critical Care Pain Observation Tool for pain (CPOT) by evaluating facial expression, body movement, muscle tension, and adherence to use of the ventilator if intubated or vocalization if extubated. Total scores range from 0 to 8, with scores higher than 2 indicating the presence of pain.
Vasopressor requirements	• Reported as proportion and median vasopressor requirements in the first 48 h after randomization
ICU and hospital LOS	• Number of calendar days (median, IQR) from randomization to discharge date from the ICU or hospital
Mortality rate	• Reported as proportion at the time of hospital discharge or 28 days after randomization, whichever comes first
Adverse events (AEs)	• Any clinically significant worsening in a study participant's condition based on clinical judgment compared to the baseline status at the time of randomization was recorded as an AE. This is applied whether or not the AE is considered to be related to the study treatment.<br><br>• Tachycardia was defined as a heart rate > 150 beats per minute<br><br>• Hyper or hypotension was classified as systolic blood pressure ≥ 180 mmHg and ≤ 90 mmHg, respectively<br><br>• Tracheostomy, and unplanned extubation (self-extubation) were reported as proportion of patients within 28 days post-randomization<br><br>• Hypersalivation: defined as frequent suctioning the first 48 h after randomization (interval between suctioning episodes 2 h or less). We calculated the modified Clinical Pulmonary Infection Score (CPIS) to differentiate secretions caused by patients' underlying lung pathology (ventilator-associated pneumonia [VAP]) vs ketamine-associated hypersalivation<br><br>• Physical restraint was reported as proportion of patients within 48 h after randomization<br><br>• The incidence of delirium was reported as proportion of patients starting on antipsychotics and positive CAM-ICU score to assess the incidence of delirium 48 h after randomization. The presence of delirium was also confirmed through a psychiatrist consultation<br><br>• We believe the administration of sedative agents is standard of practice in the ICU to minimize a patient's discomfort while on MV. Hence, the expected adverse effects will not exceed what is encountered during daily practice
Serious adverse events	• Included death or potentially life-threatening adverse effects that requires inpatient hospitalization or prolongation of hospitalization, or results in permanent or significant disability/incapacity, congenital anomaly/birth defect, or the investigator considers significant.<br><br>• An independent Research Advisory Council at our institution served as a Data Safety Monitoring Committee (DSMC) and reviewed all adverse events (including all deaths). This committee included faculty with expertise in various disciplines engaged in human subjects' research from the hospital and research centre, and also community members. ICU consultants might be invited to assist from time to time with complex issues. The committee conducted periodic reviews at the discretion of the Chair, and an expedited review is done for all serious unexpected

	adverse events (SUAEs). The committee has the authority to suspend or halt recruitment if necessary.<br><br>• No formal interim analysis of efficacy was undertaken due to possible small numbers that might preclude determination of a statistically significant difference in outcomes between the arms. No stopping rules is specified.
Recruitment rate	An average recruitment rate was calculated as the total number of recruited patients, divided by the number of study months.
Consent rate	Was defined as the proportion of patients who consented to enrolment out of those approached
Adherence rate	We defined adherence rate as receiving > 75% of protocolized intervention and assessment of protocol deviation and violation. The adherence to the protocol was assessed daily by the research team and developed as a checklist in REDCAP. Research coordinators documented all reasons for protocol non-adherence. We employed strategies to improve adherence and intervention fidelity if needed.<br><br>Calculation of consent and recruitment rate were not analysed by group but the adherence rate was assessed for each group (refer to Table 2 of main manuscript)

References:

(1) van den Boogaard M, Pickkers P, Slooter AJ, Kuiper MA, Spronk PE, van der Voort PH, van der Hoeven JG, Donders R, van Achterberg T, Schoonhoven L. Development and validation of PRE-DELIRIC (PREdiction of DELIRium in ICu patients) delirium prediction model for intensive care patients: observational multicentre study. BMJ. 2012 Feb 9;344:e420. doi: 10.1136/bmj.e420. PMID: 22323509

Table S4. Other demographic and baseline characteristics

	All (N=83)	CG (N=43)	Ketamine (N=40)	P
Other primary reason for ICU admission, N (%)				
Metabolic and endocrine disorder	1 (1.2)	1 (2.33)	0 (0)	1
Renal	6 (7.23)	3 (6.98)	3 (7.5)	1
Hematological	3 (3.61)	1 (2.33)	2 (5)	0.61
Major surgery	25 (30.1)	11 (25.6)	14 (35)	0.49
Source of infection, N (%)				
Gastrointestinal	5 (6.02)	2 (4.65)	3 (7.5)	0.67
Urine	6 (7.23)	4 (9.3)	2 (5)	0.68
Blood	9 (10.8)	3 (6.98)	6 (15)	0.30
Skin and soft tissue infections and osteomyelitis	2 (2.41)	2 (4.65)	0 (0)	0.49
Respiratory	26 (31.3)	13 (30.2)	13 (32.5)	1
Unknown	11 (13.3)	4 (9.3)	7 (17.5)	0.44
Renal replacement therapy at baseline, N (%)	7 (8.43)	3 (6.98)	4 (10)	0.71
Type of RRT, N (%)				1
iHD	5 (71.4)	2 (66.7)	3 (75)	
CVVH	1 (14.3)	1 (33.3)	0 (0)	
CVVHDF	1 (14.3)	0 (0)	1 (25)	
Urea at baseline, mmol/L	7.2 (4.25-14.4)	7.7 (4.65-17.6)	6.15 (4.12-10.9)	0.27
Mode of mechanical ventilation, N (%) [a]				0.08
AC	43 (51.8)	19 (44.2)	24 (60)	
PCV	19 (22.9)	14 (32.6)	5 (12.5)	
PS	1 (1.2)	1 (2.33)	0 (0)	
SIMV	1 (1.20)	1 (2.33)	0 (0)	
PRVC	19 (22.9)	8 (18.6)	11 (27.5)	
PH from ABG	7.31 (7.24-7.40)	7.34 (7.25-7.42)	7.3 (7.23-7.37)	0.31
PCO2 from ABG, kilopascals	6 (5-7.5)	6 (5.1-7.6)	5.9 (4.97-7.34)	0.56
PO2 from ABG, kilopascals	10.5 (7.45-14.1)	10.6 (6.85-14.1)	10.1 (8.15-14.2)	0.79
HCO3 from ABG, mmol/L	23.6 (20.1-29.2)	23.7 (20.4-31.3)	23.3 (19.5-27.5)	0.31
Metabolic acidosis, N (%)	29 (34.9)	15 (34.9)	14 (35)	1
FiO2	0.5 (0.35-0.7)	0.5 (0.4-0.7)	0.5 (0.34-0.82)	0.66

Abbreviations: AC, assist-control mode; ABG, arterial blood gas; CG, control group (donates to standard of care); CVVH, continuous veno-venous hemofiltration; CVVHDF, Continuous veno-venous hemodiafiltration; FiO2, fraction of inspired oxygen; IHD, intermittent hemodialysis; RRT, renal replacement therapy; PaO2, partial pressure of oxygen; PCO2, partial pressure of carbon dioxide; PCV, pressure control ventilation; PS, pressure support; PRVC, pressure-regulated volume control; SIMV, Synchronized intermittent mandatory ventilation

Table S5: Other safety outcomes

	All (N=83)	CG (N=43)	Ketamine (N=40)	P
28-day Tracheotomy, N (%)	22 (26.5)	11 (25.6)	11 (27.5)	1
28-day unplanned extubation/Self-extubation, N (%)	2 (2.41)	0 (0)	2 (5)	0.23
Patients who did not complete 48h of trial, N (%)	20 (24.1)	5 (11.63)	15 (37.5)	0.01
CPIS score within 48h post-randomization [a]	4 (2-5)	4 (3-6)	3.5 (2-4.25)	0.02
Pneumonia , N (%)	16 (19.3)	10 (23.3)	6 (15)	0.41
Patient started on antipsychotics within 48h post-randomization, N (%)	7 (8.43)	4 (9.3)	3 (7.5)	1
Haloperidol	1 (14.3)	1 (25)	0 (0)	1
Quetiapine	5 (71.4)	2 (50)	3 (100)	0.43
Risperidone	1 (14.3)	1 (25)	0 (0)	1
Olanzapine	1 (14.3)	0 (0)	1 (33.3)	0.43
Psychiatric physician consulted, N (%)	1 (1.20)	0 (0)	1 (2.5)	0.48
Cause of death, N (%)				
Cardiogenic shock	4 (17.4)	3 (23.1)	1 (10)	0.60
Septic shock	15 (62.5)	9 (64.3)	6 (60)	1
Hypovolemic shock	4 (17.4)	3 (23.1)	1 (10)	0.60
Respiratory	17 (68)	8 (57.1)	9 (81.8)	0.23
Metabolic	6 (26.1)	4 (30.8)	2 (20)	0.66
Multi-organ failure	16 (66.7)	11 (78.6)	5 (50)	0.20
Disposition at ICU discharge, N (%)				0.64
Morgue	28 (33.7)	16 (37.2)	12 (30)	
Floor	55 (66.3)	27 (62.8)	28 (70)	
Disposition at hospital discharge, N (%)				0.96
Home	41 (49.4)	21 (48.8)	20 (50)	
Morgue	38 (45.8)	20 (46.5)	18 (45)	
Another facility	1 (1.2)	1 (2.33)	0 (0)	
Still in hospital	3 (3.61)	1 (2.33)	2 (5)	

Data presented as n (%), mean ± sd, or median (interquartile range).

[a] CPIS score was used to differentiate secretions caused by patients' underlying lung pathology (ventilator- associated pneumonia [VAP]) vs ketamine-associated hypersalivation. Likelihood of VAP does seem to be somewhat higher when scores are >6

Abbreviations: CG, control group (donates to standard of care); CPIS, Clinical Pulmonary Infection Score ; RASS, Richmond Agitation and Sedation Scale.

Figure S2. HR and MAP at baseline, 24 hours, and 48 hours

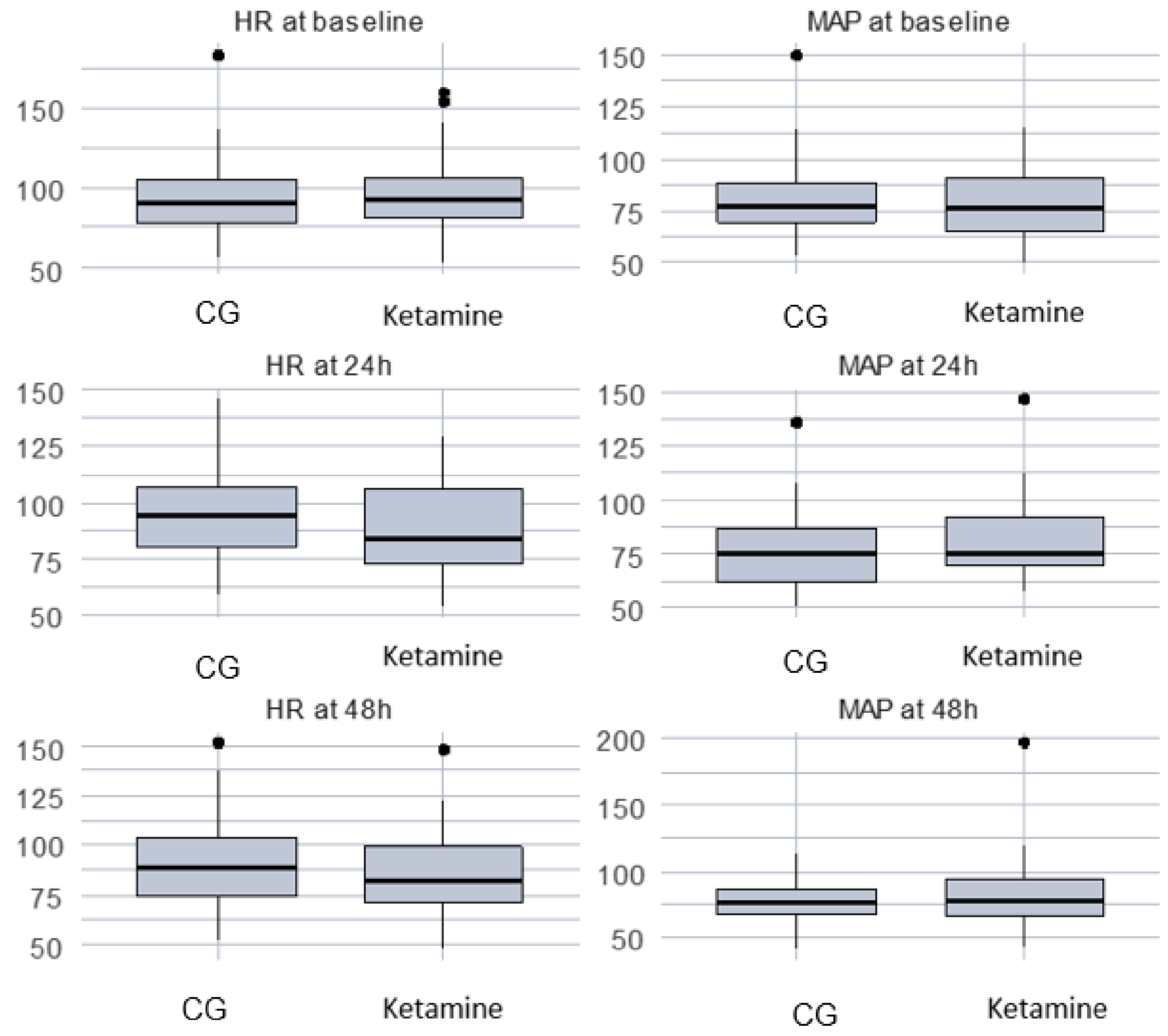

Abbreviation: CG, control group (donates to standard of care); HR, heart rate; MAP, mean arterial pressure

The middle black line is the median. The lower and upper lines of the box are the 25th and 75th percentile, respectively. The lower and upper lines represent that percentile multiplied by 1.5 the Interquartile range

Figure S3: **Subgroup analysis for selected outcomes**

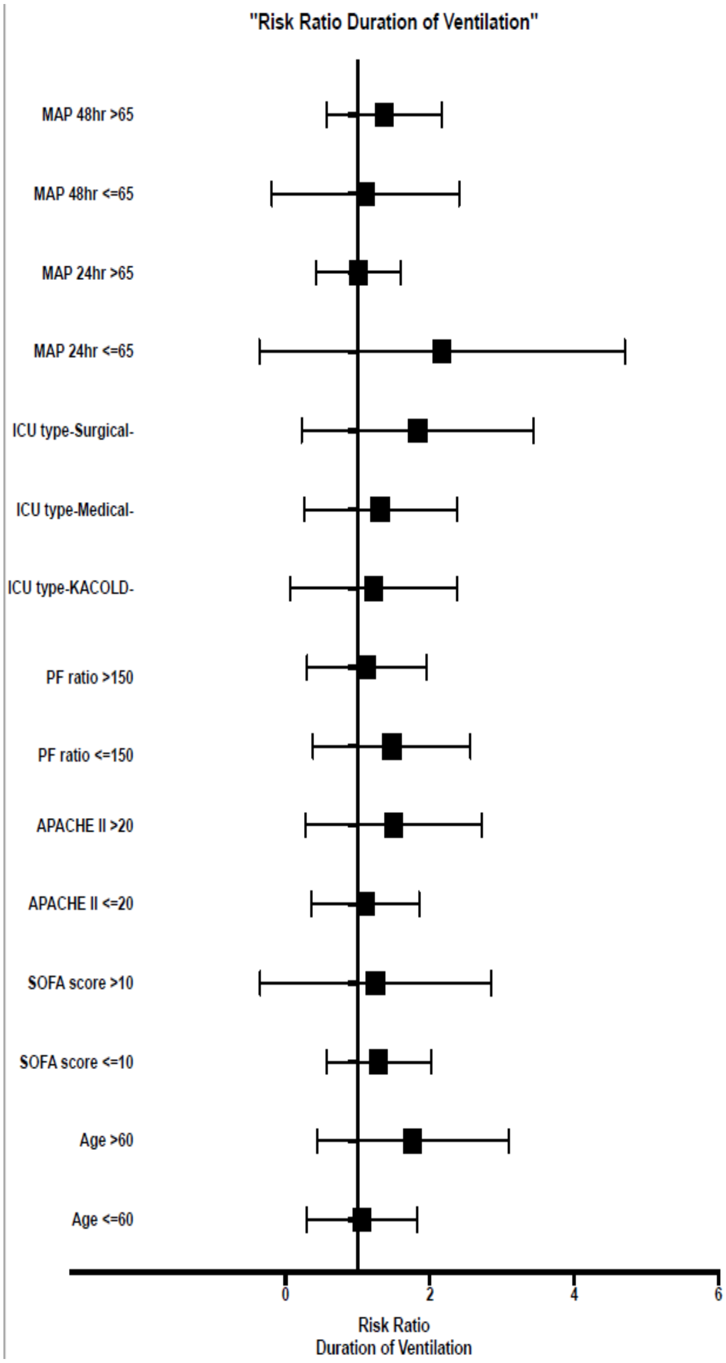

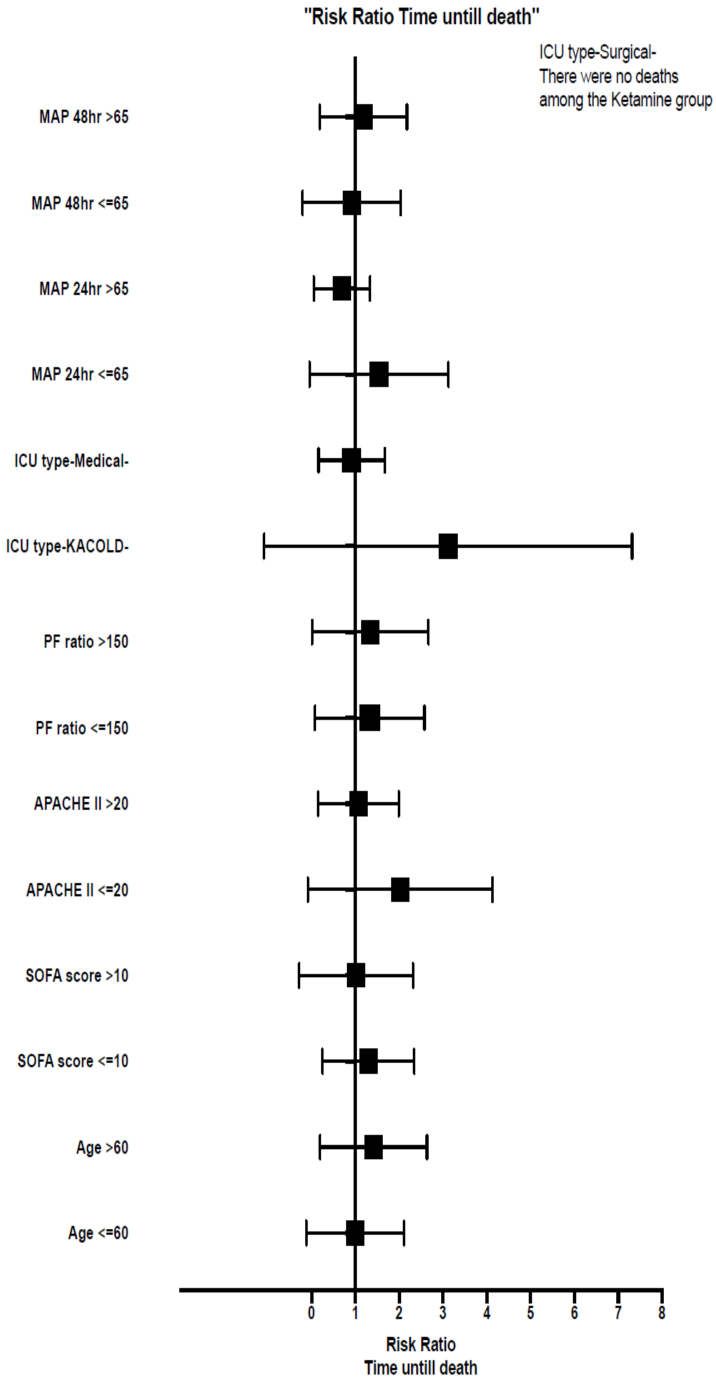

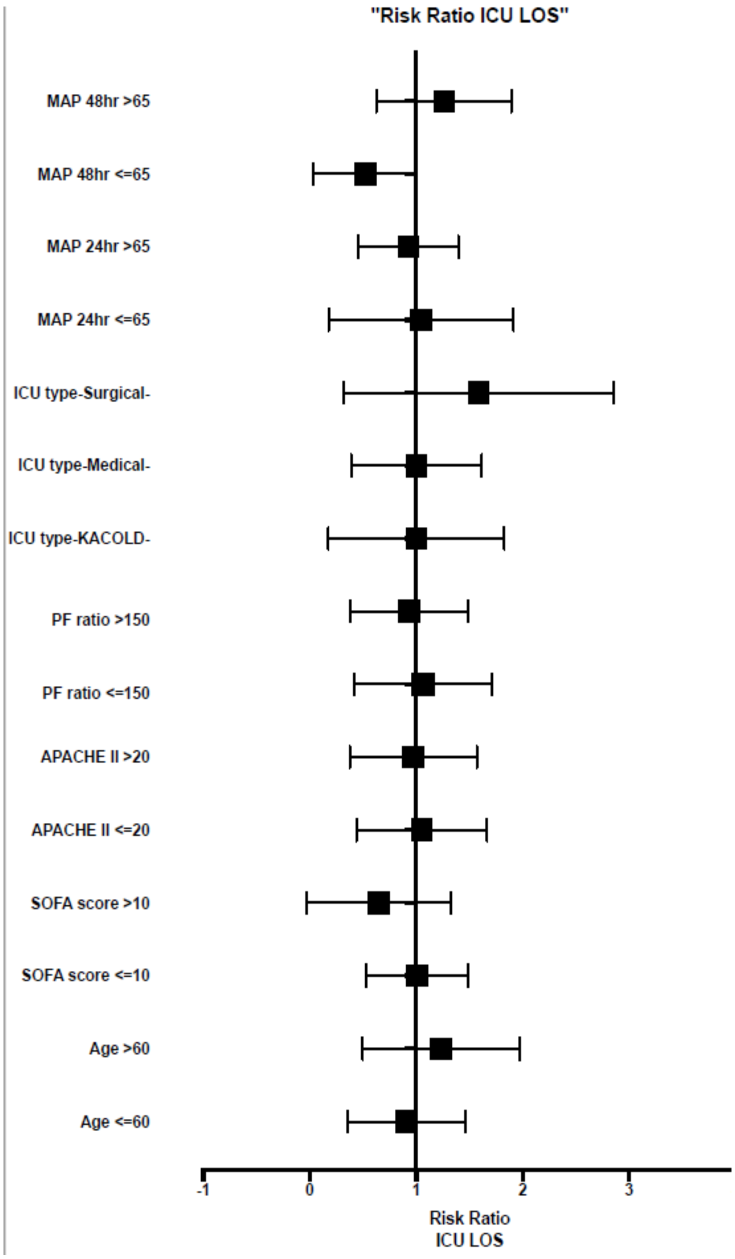

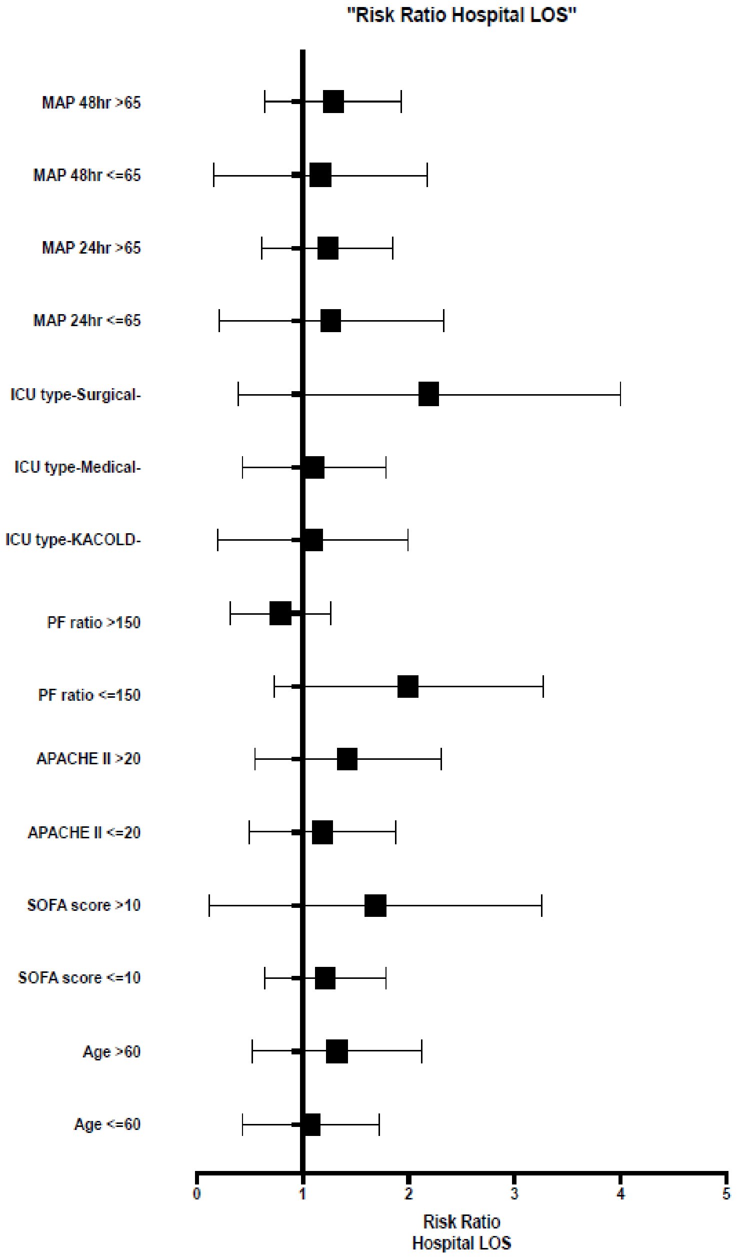

KACOLD donates to transplant ICU

The results of subgroup analysis should be interpreted with caution due to the small sample size, wide and overlapping confidence interval. It is generally recommended that feasibility and pilot studies descriptively evaluate a trial's feasibility, acceptability and safety rather than test the effectiveness hypotheses of the planned main large-scale trial. Thus, robust and rigorous assessment of the subgroup of patients will be explored further in adequately sized definitive trial.

Table S6: Proportion of sedatives and vasopressors

	Baseline				48h post-randomization			
	**All (N=83)**	**CG (N=43)**	**Ketamine (N=40)**	**P**	**All (N=83)**	**CG (N=43)**	**Ketamine (N=40)**	**P**
Fentanyl, N (%)	80 (96.4)	40 (93)	40 (100)	0.24	82 (98.8)	43 (100)	39 (97.5)	0.48
Propofol, N (%)	70 (84.3)	35 (81.4)	35 (87.5)	0.64	48 (57.8)	24 (55.8)	24 (60)	0.87
Midazolam, N (%)	47 (56.6)	24 (55.8)	23 (57.5)	1	14 (16.9)	8 (18.6)	6 (15)	0.89
Dexmedetomidine, N (%)	.	.	.		16 (19.3)	12 (27.9)	4 (10)	0.05
Norepinephrine, N (%)	50 (60.2)	26 (60.5)	24 (60)	1	52 (62.7)	27 (62.8)	25 (62.5)	1
Epinephrine, N (%)	6 (7.23)	4 (9.3)	2 (5)	0.68	5 (6.02)	1 (2.33)	4 (10)	0.19
Phenylephrine, N (%)	24 (28.9)	16 (37.2)	8 (20)	0.14	11 (13.3)	5 (11.6)	6 (15)	0.89
Vasopressin, N (%)	8 (9.64)	5 (11.6)	3 (7.5)	0.71	15 (18.1)	8 (18.6)	7 (17.5)	1
Dopamine, N (%)	4 (4.82)	2 (4.65)	2 (5)	1	5 (6.02)	3 (6.98)	2 (5)	1

Data presented as n (%),

CG, control group and donates to standard of care

Table S7: Sensitivity analysis for sedatives and vasopressors requirements excluding patients started on NMB post-randomization

	CG (N=41)	Ketamine (N=35)	P
Patient on fentanyl within 48h, N (%)	41 (100)	34 (97.1)	0.46
Cumulative use of fentanyl (μg) 48h post-randomization	3817 (2200-5900)	3400 (1500-6298)	0.87
Cumulative use of fentanyl (μg /Kg) 48h post-randomization	66.1 (32.7-103)	61.4 (19.5-108)	0.91
Patient on propofol 48h post-randomization, N (%)	22 (53.7)	20 (57.1)	0.94
Cumulative propofol use (mg) 48h post-randomization	2161 (398-3406)	1688 (778-4272)	0.88
Propofol cumulative use (mg/kg) 48h post-randomization	33.6 (7.65-58.5)	27.8 (9.62-56.7)	0.76
Patient on midazolam within 48h post-randomization, N (%)	7 (17.1)	4 (11.4)	0.71
Cumulative use of midazolam (mg) 48h post-randomization	6 (4- 54.5)	58.5 (12.5-110)	0.29
Midazolam cumulative use (mg/kg) 48h post-randomization	0.21 (0.09-0.72)	0.74 (0.19-1.43)	0.45
Dexmedetomidine within 48h post-randomization, N (%)	12 (29.3)	4 (11.4)	0.11
Cumulative use of dexmedetomidine (μg) 48h post-randomization	667 (357-1222)	711 (310-1730)	0.90
Cumulative use of dexmedetomidine (μg /Kg) 48h post-randomization	9.34 (5.33-22)	18 (4.67- 35.5)	0.63
Patient on norepinephrine within 48 h, N (%)	25 (60.89)	21 (60)	1
Cumulative use of norepinephrine (mg) 48h post-randomization	8.07 (5.27 - 20.07)	8.21 (3.66-28)	0.97
Patient of epinephrine within 48h post-randomization, N (%)	1 (2.44)	2 (5.71)	0.59
Cumulative use of epinephrine (mg) 48h post-randomization	29.2 (29.2- 29.2)	3.805 (1.52 - 6.09)	0.22
Patient on phenylephrine within 48h post-randomization, N (%)	5 (12.2)	5 (14.29)	1
Cumulative use of phenylephrine (mg) 48h post-randomization	36 (0.3-72.55)	0.6 (0.213-81.3)	0.75
Patient on dopamine within 48h post-randomization, N (%)	3 (7.32)	2 (5.71)	1
Cumulative use of dopamine (mg) 48h post-randomization	562.56 (401.74 - 675.84)	602.3 (489.6- 715)	0.56
Patient on vasopressin within 48h post-randomization, N (%)	7 (17.07)	5 (14.29)	1
Cumulative use of vasopressin (units) 48h post-randomization	48 (21.6 - 95.57)	69.6 (49.2-104.4)	0.41

Data presented as n (%), mean ± sd, or median (interquartile range). CG, control group and donates to standard of care

Table S8: Post-hoc sensitivity analysis excluding patients who did not complete 48-hours due to extubation or sedation weaned off [a]

**Variable**	**CG (N=38)**	**Ketamine (N=25)**	***P***
Duration of mechanical ventilation, mean (SD), days	9 (5-24)	10 (7-14.2)	0.24
Ventilation-free days, median (IQR), days [b]	16.5 (0-24)	19 (0-21.5)	0.61
Discharge from ICU, N (%)	35 (92.11)	23 (92)	0.98
ICU length of stay, median (IQR), days	13 (7.75- 25.5)	17 (12-27)	0.3
Hospital discharge, N (%)	36 (94.7)	24 (96)	0.8
Hospital length of stay, median (IQR), days	30.5 (13-50.5)	26 (18.5-38)	0.9
Fentanyl 48h post-randomization, N (%)	38 (100)	25 (100)	.
Fentanyl cumulative use (µg) 48h post-randomization, median (IQR)	3925 (2230-6410)	6000 (2445.5-8650)	0.06
Propofol 48h post-randomization, N (%)	23 (60.5)	17 (68)	0.55
Propofol cumulative use (mg) 48h post-randomization, median (IQR)	2132 (540-3452)	3550 (1428-5880)	0.35
Midazolam 48h post-randomization, N (%)	7 (18.42)	4 (16)	0.80
Midazolam cumulative use (mg) 48h post-randomization, median (IQR)	7 (5.25-76.2)	78.2 (21.4-175)	0.136
Dexmedetomidine 48h post-randomization, N (%)	11 (29)	2 (8)	0.08
Dexmedetomidine cumulative use (µg) 48h post-randomization, median (IQR)	758 (339-1228)	1947 (988-2905)	0.84
Norepinephrine 48h post-randomization, N (%)	23 (60.5)	19 (76)	0.2
Norepinephrine cumulative use (mg) 48h post-randomization, median (IQR)	9.7 (6.7 – 27.9)	7.6 (3.3-27.59)	0.5
Epinephrine 48h post-randomization, N (%)	0 (0)	2 (8)	0.1
Epinephrine cumulative use (mg) 48h post-randomization, median (IQR)	-	1.8 (1.5-2)	-
Phenylephrine 48h post-randomization, N (%)	5 (13.16)	3 (12)	0.9
Phenylephrine cumulative use (mg) 48h post-randomization, median (IQR)	36 (0.3-72.55)	0.125 (0.1-0.3)	0.13
Dopamine 48h post-randomization, N (%)	1 (2.63)	1 (4)	0.8
Dopamine cumulative use (mg) 48h post-randomization, median (IQR)	675.8 (675.8 - 675.8)	715 (715- 715)	0.3
Vasopressin 48h post-randomization, N (%)	7 (18.42)	4 (16)	0.8

Vasopressin cumulative use (units) 48h post-randomization	46.8 (20.7 - 95.57)	89.6 (59.2-109.8)	0.2

Data presented as n (%), mean ± sd, or median (interquartile range).

[a] This sensitivity analysis was conducted to address whether the discrepancy in numbers of patients who did not complete 48h of trial (due to extubation and sedation weaned off) would contaminate the interpretation of the results of this pilot study. This analysis included per-protocol population, defined as mITT population after exclusion of subjects who did not complete 48-hours post-randomization. The results were consistent with primary analysis.

[b] VFDs were calculated by subtracting number of ventilation days from 28 after assigning VFD=0 for patients who died during 28 days.

CG, control group and donates to standard of care.

Table S9: Ketamine studies that showed and did not show opioid-sparing effect

Study and design	Study population
Studies that ***did not show*** Opioid-sparing effect	
Perbet *et al* Single center RCT	Including 40% patients with acute respiratory failure. Compared remifentanil and low-dose ketamine with remifentanil and placebo, and failed to show any opioid sparing effect
Alshahrani MS, *et al.* RCT	Sickle cell disease patients (not on MV) in emergency department randomized in a 1:1 ratio to receive either a single dose low-dose ketamine (0.3 mg/kg) or a single dose morphine (0.1 mg/kg). Ketamine use compared to morphine was associated with similar reduction in pain score NPRS and use of rescue opioid analgesia (MD 0.74 mg morphine equivalent; 95% CI -0.36 to 1.84)
ATTAINMENT trial. RCT	ICU adult patients on MV (50% medical ICU, 26.5% surgical ICU, and 25.3% transplant ICU; median SOFA 8, and APACHE II 20, 60 % with ARDS)
Studies that ***showed*** Opioid-sparing effect and reduction of sedatives	
Guillou *et al* (RCT)<br>Ketamine vs. placebo	Adult surgical ICU patients following major abdominal surgery on morphine PCA.
Pruskowski *et a*l (RR): Pre- and post-ketamine initiation	Adult trauma patients on MV
Buchheit *et al* (RR): Pre- and post-ketamine initiation	ICU patients on MV on SBT period
Groetzinger *et al* (RR) Pre- and post-ketamine initiation	Adult MV patients (59% MICU , 20% transplant ICU , 15% trauma ICU)

RCT = randomized controlled trial, RR = retrospective review

References :

1. Perbet S, Verdonk F, Godet T, Jabaudon M, Chartier C, Cayot S, Guerin R, Morand D, Bazin JE, Futier E, Pereira B, Constantin JM (2018) Low doses of ketamine reduce delirium but not opiate consumption in mechanically ventilated and sedated ICU patients: a randomised double-blind control trial. Anaesth Crit Care Pain Med 37:589–595
2. Alshahrani, M.S., Asonto, L.P., El Tahan, M.M. et al. Study protocol for a randomized, blinded, controlled trial of ketamine for acute painful crisis of sickle cell disease. Trials 20, 286 (2019). https://doi.org/10.1186/s13063-019-3394-4
3. Alshahrani, M.S., Alsulaibikh A.H., ElTahan, M.M. et al. Ketamine Use for Acute Painful Crisis of Sickle Cell Disease: A Randomized Controlled Trial (research forum abstract). Ann Emerg Med 2019; 74(4): S89-90
4. Guillou N, Tanguy M, Seguin P, et al: The effects of small-dose ketamine on morphine consumption in surgical intensive care unit patients after major abdominal surgery. Anesth Analg 2003; 97:843–847
5. Pruskowski KA, Harbourt K, Pajoumand M, et al: Impact of ketamine use on adjunctive analgesic and sedative medications in critically ill trauma patients. Pharmacotherapy 2017; 37:1537–1544
6. Buchheit JL, Yeh DD, Eikermann M, et al: Impact of low-dose ketamine on the usage of continuous opioid infusion for the treatment of pain in adult mechanically ventilated patients in surgical intensive care units. J Inten Care Med 2019; 34:646–651
7. Groetzinger LM, Rivosecchi RM, Bain W, et al: Ketamine infusion for adjunct sedation in mechanically ventilated adults. Pharmacotherapy 2018; 38:181–188

Figure S4: Proposed treatment algorithm for the definitive trial with modified ketamine dosing regimen

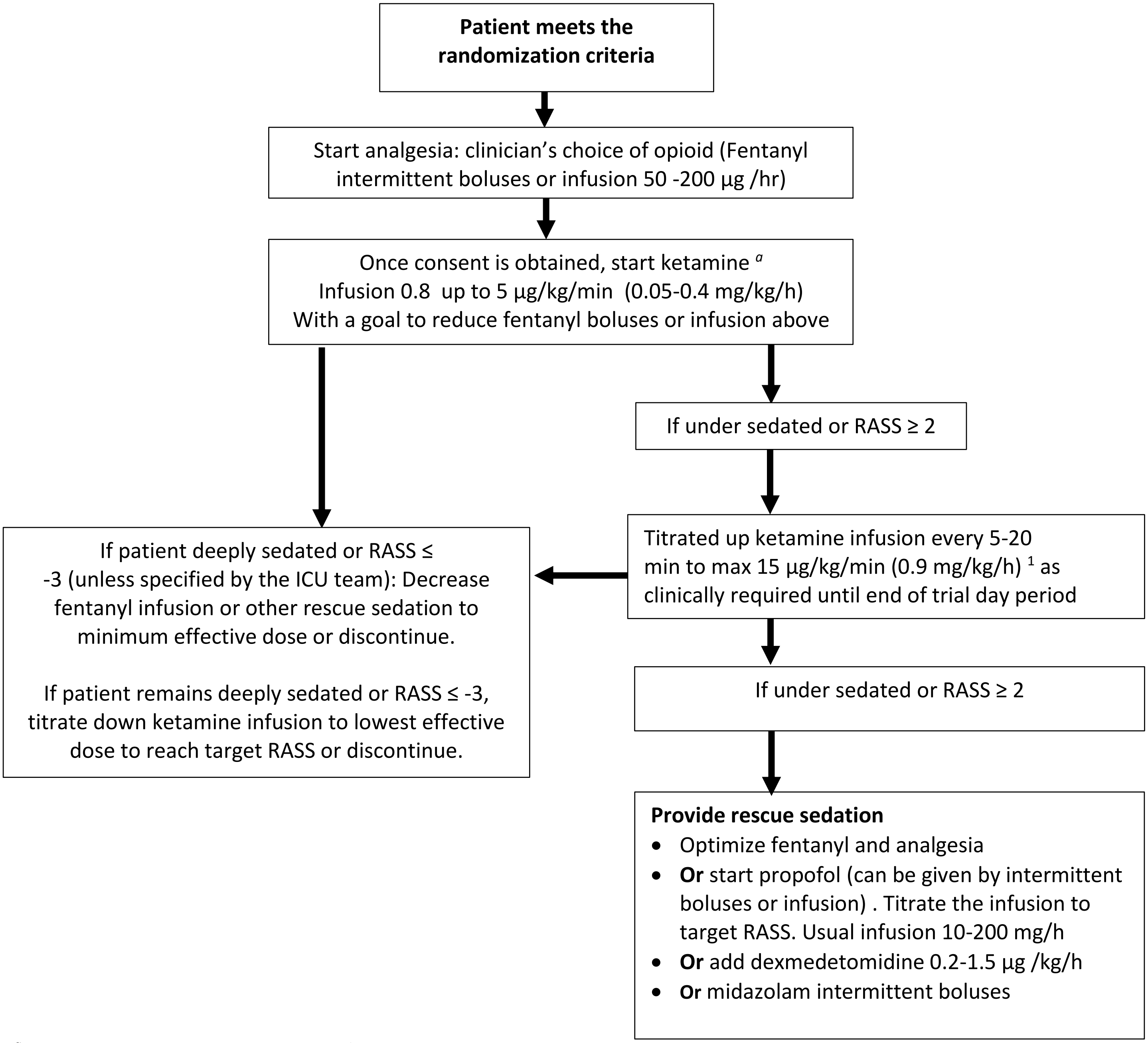

[a] Consider a loading dose if 0.1-0.5 mg/kg

**Titrating Up**	**Titrating Down**
□ Initiation □ Restart after SAT<br>□ Undersedation □ Starting paralytics post-randomization	□ Oversedation □ Side effect<br>□ During SAT □ Off MV and no sedation required<br>□ Patient went to OR □ Off paralytics post-randomization

Discontinue drug permanently if: Coma due to new CNS insult, severe allergic reaction , severe tachyarrhythmia or hypertension, severe agitation or hallucination, withdraw from trial enrollment , end of trial day period

Reference: (1) Hurth KP, Jaworski A, Thomas KB, Kirsch WB, Rudoni MA, Wohlfarth KM: The reemergence of ketamine for treatment in critically ill adults. Crit Care Med 2020; 48:899-911
